# The Effects of Brodalumab on the Fungal Microbiome in Patients with Psoriasis

**DOI:** 10.3390/ijms251910239

**Published:** 2024-09-24

**Authors:** Admir Vižlin, Ajša Bajramović, Ylva Andersch Björkman, Yadhu Kumar, Maria Göthe, Martin Gillstedt, Amra Osmančević

**Affiliations:** 1 Department of Dermatology and Venereology, Institute of Clinical Sciences, Sahlgrenska Academy, University of Gothenburg, 413 45 Gothenburg, Sweden; admir.vizlin@gmail.com (A.V.);; 2 Faculty of Medicine, Sarajevo School of Science and Technology (SSST), 71000 Sarajevo, Bosnia and Herzegovina; ajsabajramovic47@gmail.com; 3 Department of Dermatology and Venereology, Sahlgrenska University Hospital, Region Västra Götaland, 413 45 Gothenburg, Sweden; 4 Eurofins Genomics, 78467 Konstanz, Germany

**Keywords:** psoriasis, brodalumab, gut microbiome, fungi, TNF-alpha inhibitors

## Abstract

The gut microbiota plays a critical role in immune system function, with dysbiosis linked to systemic inflammation, contributing to conditions like psoriasis and depression. Although biological treatments for severe psoriasis are known to impact gut bacteria, less is understood about their effects on fungi. This study aims to investigate fungal gut microbiota changes in psoriasis patients transitioning from TNF-α inhibitors to brodalumab. Fecal samples from 20 patients were analyzed using Illumina MiSeq sequencing of the ITS2 region of 18S rRNA. Microbial diversity was assessed through Bray–Curtis dissimilarity and the Shannon–Wiener index. Clinical outcomes were measured using clinical scores for psoriasis and depression severity, with statistical analysis performed via Wilcoxon signed-rank tests and PERMANOVA. Results showed that Ascomycota was the dominant fungal phylum in both treatment groups, with *Saccharomyces*, *Penicillium*, *Candida*, and *Debaryomyces* as prevalent genera. No significant changes occurred at the phylum level after switching to brodalumab, though minor genome-level variations were observed. Beta diversity analysis highlighted inter-patient variability, with no significant correlation between fungal composition and clinical outcomes. Despite improved clinical scores, the fungal gut microbiota remained largely stable, suggesting that brodalumab does not significantly alter fungal communities in psoriasis patients. Further research is needed for a comprehensive understanding.

## 1. Introduction

Bacteria have been well established to be the primary constituents of a healthy gut microbiome; as such, not a lot of attention has been given to the other inhabitants, the fungi. Part of the reason for this is the added difficulty in analyzing the stool samples for fungi. The main area of interest in identifying specific fungal species is the ‘internal transcribed spacer region’ (ITS). The ITS varies in length between fungal species but the variation is not as pronounced which makes it difficult to distinguish between them. Additionally, there is a lack of reliable reference databases for classifying fungal sequences [1].

There is great variability among fungal species in different samples. Bray–Curtis dissimilarity (0.0–1.0, where 1 means maximum dissimilarity) can be used to examine the myriad of microbes that are shared between two samples and their number found in each. Fungal communities show a great intra- and inter-volunteer dissimilarity, with a Bray–Curtis dissimilarity approaching 1.0 [2].

Despite the great variability, studies show that certain fungi do appear more commonly than others, including *Saccharomyces*, *Malassezia*, and *Candida*, with *S. cerevisiae*, *M. restricta*, and *C. albicans* operational taxonomic units (OTUs) present in 96.8%, 88.3%, and 80.8% of samples [2]. *Malassezia* species, common on skin, nails, and hair, are implicated in various dermatological conditions [3]. As of this day, *Candida* is the most common fungal organism found in the human gut microbiome [4].

Gut dysbiosis is implicated in various inflammatory conditions including both psoriasis and depression. Fungal dysbiosis in depressed patients is attributed to the impact of the inter-relationship between fungi and bacteria. A study found that depressed patients have increased levels of certain fungi (e.g., *Candida*, *Chaetomium*, *Neocosmospora*, *Occultifur*, *Neocosmospora*, *Clonostachys*, and *Chaetomium*) and decreased levels of others (e.g., *Scedosporium*, *Purpureocillium*, *Penicillium*, *Clonostachys*, and *Aureobasidium*) [5]. The most influential dysbiosis is seen with the increase in *C. albicans*. It is believed that the mucocutaneous pathobionts of *C. albicans* induce and activate the human antifungal T helper 17 (Th17) cells. These Th17 cells then enter the peripheral circulation where they eventually cross the blood-brain barrier (BBB). A recent study has proposed that Th17 cells enter and cross the BBB by using a dual immunoglobulin domain-containing cell adhesion molecule (DICAM) [6]. Once in the brain, they activate the hippocampal microglia which results in the release of interleukin (IL)17 and IL-1β [5]. Gliotoxin from *Candida* and *Aspergillus* species also crosses the BBB, destroying astrocytes and oligodendrocytes, leading to immune reactions and depressive symptoms [7]. Analysis of blood samples from depressed patients showed higher concentrations of IL-17 and IL-23 when compared to controls, and a decreased amount of IL-21 and IL-35 [8]. 

Research on fungal dysbiosis in psoriasis patients has mostly focused on the skin microbiota, rather than gut microbiota. As such, very little is known about the kind of fungal species inhabiting the gut of psoriatic patients. Psoriasis pathogenesis involves multiple immune cells (leukocytes, macrophages, neutrophils, keratinocytes, and T cells) releasing proinflammatory factors like IL-1, IL-6, tumor necrosis factor (TNF)α, IL-17A, IL-22, and IL-23. The dendritic cells are the ones initiating the immune cascade for psoriatic inflammation. Firstly, they bind to LL-37 on damaged keratinocytes, which activates the dendritic cell. Once activated, production of TNF-α soon follows which will make further complexes with LL-37. The TNF-α/LL-37 complex aids in dendritic cell maturation and its subsequent production of IL-12 and IL-23. These two interleukins promote Th17 maturation to produce IL-17, which then stimulates keratinocyte proliferation and the production of neutrophil recruiting factors (chemokine (C-X-C motif) ligand (CXCL)1/2/3/5/8). As such, IL-23 and IL-17 can be considered the most important cytokines for psoriasis pathogenesis [9]. A study has shown that the severity of psoriasis is related to the amount of IL-23/IL-17 and their ability to influence keratinocyte proliferation [10]. 

In the gut, the Mincle-Syk axis, along with innate lymphoid cells (ILC3s) and Th17 cells, regulates IL-17 and IL-23 levels to maintain intestinal barrier integrity [11]. When gut microbiome dysbiosis occurs, the integrity of the barrier is disrupted due to the change in the level of microbial metabolites (short-chain fatty acids, secondary bile acids, tryptophan, lipopolysaccharides (LPSs) and phenols). Th17 cells require fatty acid synthesis for their maturation and are directly influenced by the resident T regulatory cells for their inhibitions. Dysbiosis also leads to the inactivation of Treg cells due to the inhibition of maturation coming from hypoxic conditions. As such, a shift in the balance is observed with Th17 and T regulatory cells (Treg), (Shift to Th17). This produces more IL-17 and IL-23, thereby directly influencing the systemic inflammation and its subsequent psoriatic pathogenesis [12]. 

When dealing with inflammatory conditions, biological agents are the treatment of choice. Brodalumab is a human anti-IL-17RA monoclonal IgG2 antibody that binds to the IL-17 receptor and directly inhibits it. This is unlike other IL-17 inhibitors which only inhibit the IL-17A and/or IL-17F molecules.

Multiple studies have shown brodalumab’s effectiveness in reducing psoriatic lesions [13,14]. However, there are conflicting reports on its impact on mental health; some studies report suicidal ideation and behavior as side effects [15], while others show improvements in depression and anxiety compared to a placebo in treating plaque psoriasis [16]. Despite these conflicting results, psoriasis is consistently treated. TNF- α inhibitors prevent TNF from aiding in the maturation of Th17 cells, thereby indirectly reducing the levels of IL-17. A study comparing TNF-α inhibitors and brodalumab for psoriasis treatment found improvement in depressive symptoms with brodalumab but insufficient response with TNF-α inhibitors [17]. Psoriasis can be assessed using the Psoriasis Area and Severity Index (PASI) and the Dermatology Life Quality Index (DLQI). A study noted that when treating plaque psoriasis, a reduction in PASI of at least 75% translates to significant quality-of-life improvement [18]. According to the Swedish guidelines, severe psoriasis is defined as PASI ≥ 10 and DLQI ≥ 10, moderate psoriasis PASI 3–9 and DLQI 6–9, and mild as PASI ≤ 3 and DLQI ≤ 5 [19]. An assessment of depressive symptoms can be made using the Montgomery–Åsberg Depression Scale–Self-Administered (MADRS-S) [20] and the Hospital Anxiety and Depression Scale (HADS) [21]. 

## 2. Results

### 2.1. Demographics

A total of 22 patients were initially selected from the SAHL1011 study [17], of which 20 (14 men and 6 women) met the inclusion criteria. There were 3 patients who did not complete the study due to joint pain (*n* = 1), travel limitations (*n* = 1), or stomach issues related to TNF-α inhibition (*n* = 1). Most responded to brodalumab treatment, showing significant improvements in psoriasis severity (PASI, *p* < 0.0001), quality of life (DLQI, *p* = 0.004), and depressive symptoms (MADRS-S, *p* = 0.0007) [17]. See Table 1.

### 2.2. Raw Data

Eurofins Genomics (Konstanz, Germany) performed data sequencing targeting the 18s rRNA ITS2 region. The dataset had a total of 114,540 to 160,000 reads per sample, where the average proportion of high-quality reads ranged from 62% to 94%. Sequence reads were further assembled to generate amplicon length sequences. With a minimum of 60,000 amplicon readouts per sample, the final amplicon reads had an average length of 348 base pairs. Sequence quality remained consistent across all samples. 

### 2.3. Composition of the Gut Microbiota

**Figure 1:**
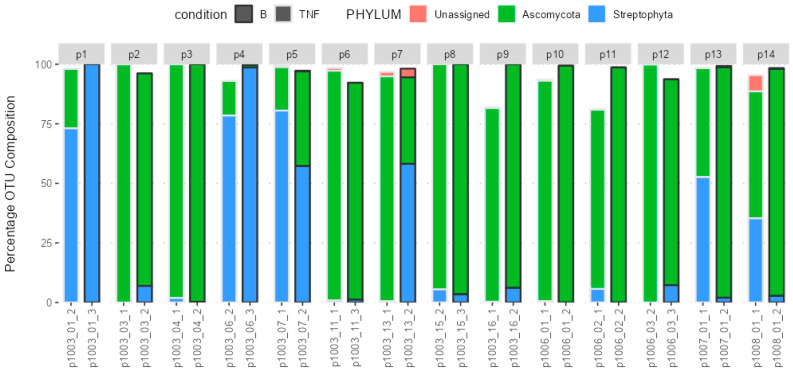
The bar chart presents the composition of the gut microbiota on the phylum level before (TNF) and after (B) the switch to brodalumab. Each number represents one patient (p(n)). Each bar is a sample. The *y*-axis represents the relative abundance of phyla given as OTU composition. Five subjects did not submit samples after the switch, thus excluding them from the comparative analysis (total of 14 patients remained in the final analysis).

The fungal gut microbiota mainly belonged to the Ascomycota phylum. Figure 1 depicts the microbiota profile in patients with psoriasis pre- and post-brodalumab switch.

**Figure 2:**
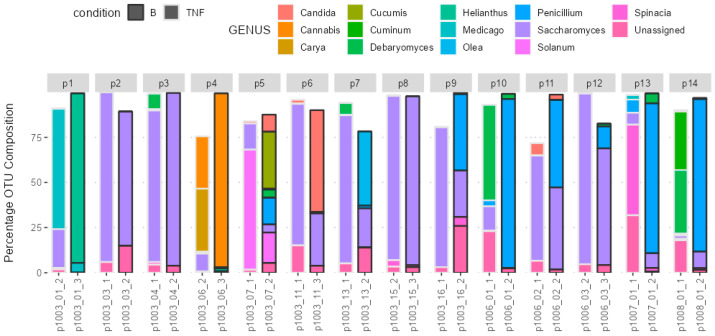
The bar chart presents the composition of the gut microbiota on the genus level before (TNF) and after (B) the switch to brodalumab. Each number represents one patient (p(n)). Each bar is a sample. The *y*-axis represents the relative abundance of phyla given as OTU composition.

The fungal gut microbiota mainly belonged to the *Saccharomyces*, *Penicillium*, *Candida*, and *Debaryomyces* genera. Figure 2 depicts the microbiota profile in patients with psoriasis pre- and post-brodalumab switch.

### 2.4. Difference in Groups before and after the Treatment Switch

#### Principal Coordinate Analysis

**Figure 3:**
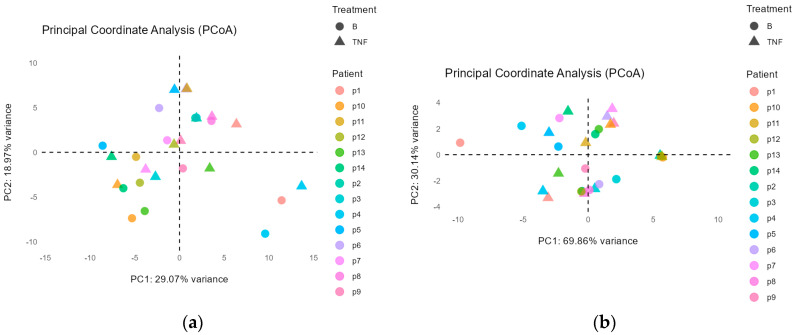
PCoA at the genus (**a**) and phylum (**b**) level. Patients are represented as specific colors, while the condition before the switch (TNF) is represented as a triangle and the condition after the switch (B) is represented as a circle. The distances between points (shapes) reflect the dissimilarity between microbial communities.

The principal coordinate analysis (PCoA) showed that there were no significant differences in fungal microbiota after switching to brodalumab at the phylum level (*p* = 0.74) (Figure 3a); however, at the genus level there is a small significant difference (*p* = 0.028) (Figure 3b). Between patients, microbiota composition was significantly different at both the phylum (*p* = 0.02) (Figure 3a) and the genus level (*p* = 0.008) (Figure 3b). After 12 weeks, the treatment switch did cause slightly significant compositional changes at the genus level, but not at the phylum. It is assumed that the individual microbiota fungal composition remains unique and significantly different.

### 2.5. Beta Diversity Analysis

**Figure 4:**
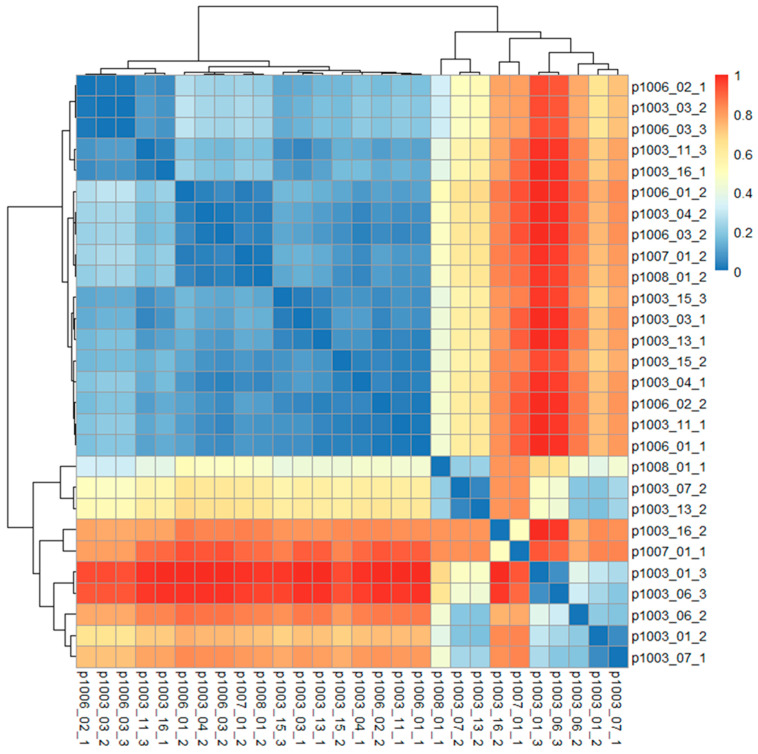
Heat map of the beta diversity of fungi phyla. Red represents a value of 1, high dissimilarity between samples; blue represents a value of 0, low dissimilarity between samples.

Beta diversity analysis with a heat map showed that there were no significant differences between the phyla composition pre- and post-brodalumab switch. Some samples exhibited high inter-volunteer dissimilarity, see Figure 4.

### 2.6. Alpha Diversity Analysis

**Figure 5:**
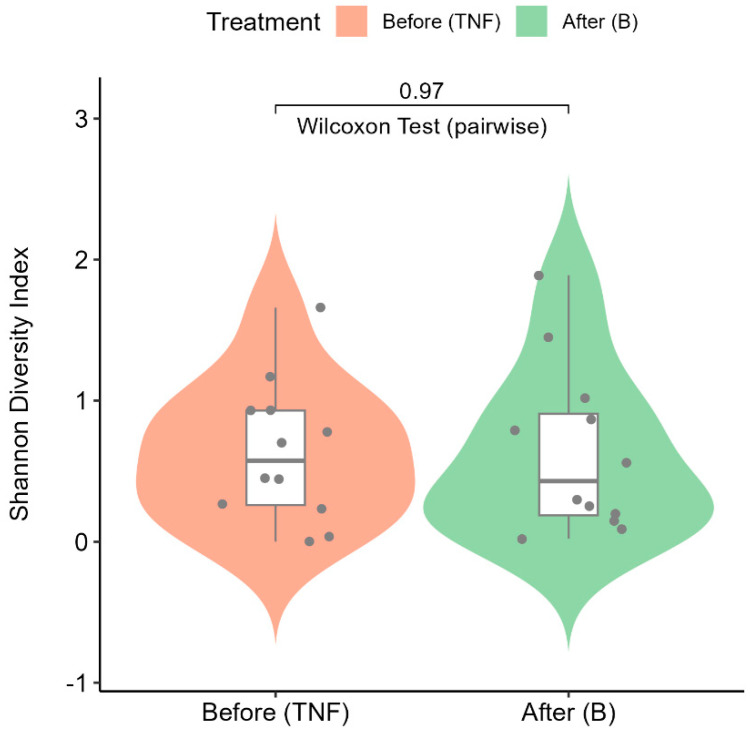
Violin plot showing the distributions of the Shannon–Wiener diversity indices across treatment groups. The pink violin represents diversity indices from patients before treatment (TNF), while the green violin represents diversity indices after treatment (B).

The Shannon–Wiener diversity index (alpha diversity) is calculated to quantify the diversity of microbial communities in patient groups before and after treatment. The violin plot (Figure 5) shows the distributions of the Shannon–Wiener diversity indices across treatment groups. The alpha diversity values are consistent between the treatment groups, as evidenced by the non-parametric pairwise statistical test (Wilcoxon signed rank test) performed on the two groups. The *p*-value of 0.97 indicates that no significant differences were observed due to treatment on microbial compositions before and after treatment.

### 2.7. Correlation to Clinical Variables

After correcting for multiple testing there was no significant correlation (*p* = 1.00) of fungi phyla and genera to the clinical variables PASI, DLQI, MADRS-S, and HADS (Table 2). Based on the values of clinical variables, specific phyla and genera remain unchanged and, as such, are also unaffected by the variable change.

## 3. Discussion

The fungal gut microbiota predominantly belonged to the Ascomycota phylum and the following genera: *Saccharomyces*, *Penicillium*, *Candida* and *Debaryomyces*. After 12 weeks, the treatment switch to brodalumab did not result in significant compositional changes at the phylum level. Ascomycota remained the dominant phylum throughout this study. However, at the genus level, there were minor but statistically significant compositional changes (*p* = 0.028), indicating some alterations in the gut microbiota profile. We believe this is due to plant reads, as seen from Figure 2; their presence in the fecal composition overrode the fungal reads and resulted in statistical inaccuracy. Nevertheless, given this minimal level of significance and the absence of notable microbiome changes at the phylum level, it can be concluded that the fungal microbiota remained largely unchanged following the treatment switch to brodalumab over the 12-week period. 

Studies by Fry & Baker (2007) and Waldman et al. (2001) report *Candida* to be the main gut inhabitant of the microbiome of untreated psoriasis patients [22,23]. Our study reports *Candida* to be dominant only in a few patients, and it is not the dominant genera overall sample-wise. It is interesting to note that Yeung et al. (2022) reported that *Candida* skin infections occur more commonly following brodalumab treatment [24]. When considering the healthy gut microbiome, Hoffmann et al. (2013) reported that *Candida* and *Saccharomyces* are among the most frequently detected fungal genera [25]. This can suggest that the general fungal composition may not differ drastically from healthy individuals at the genus level, but the roles these fungi play in a psoriatic context may be distinct due to the underlying inflammatory environment. Existing research supports this notion, where the bacterium *Serratia marcescens* increases the levels of *C. albicans* in Crohn’s disease, while it has antifungal properties through VI secretion system proteins, Tfe1 and Tfe2, in healthy individuals [26,27]. 

Studies like those by Nash et al. (2017) have highlighted that fungal communities exhibit high intra- and inter-volunteer dissimilarity (approaching 1.0) [2]. This aligns with our finding that the fungal microbiota was statistically different at both the genus and phylum level between patients, as shown by Bray–Curtis dissimilarity heat map in Figure 4. This was expected, since this variability could be driven by factors such as diet, genetics, environmental exposures, and the specific characteristics of each patient’s psoriasis. Our findings and previous research suggest that individualized treatment approaches may be necessary to account for the potential differences in response to brodalumab treatment, in part due to this variance in fungal communities. 

This could explain some conflicting findings. A study followed 6243 psoriatic patients and noted that, after brodalumab, there were 6 completed suicides, 11 suicide attempts and 22 suicidal ideations [15], whereas another study showed an improvement in depression and anxiety in patients who took brodalumab compared to a placebo group for treatment of plaque psoriasis [16]. There is an absence of a significant correlation between fungal microbiota composition and clinical variables (PASI, DLQI, MADRS-S, HADS). Similarly, a previous study did not find any alteration in bacterial microbiome in the same group of patients [28]. This indicates that the relationship between gut microbiota and clinical outcomes in psoriasis may be more complex and not solely dependent on the presence or abundance of specific microbial taxa, or that not enough time has passed for change to take place. PASI and DLQI scores did significantly decrease after switching to brodalumab. Studies report a PASI score improvement anywhere between 75–87% with brodalumab 210 mg [13,14,16]. 

While thorough in its methodology, the study has several limitations that could impact its findings. First, as a pilot project, the study’s small sample size may limit the generalizability of the results and reduce statistical power. Furthermore, this study did not address whether there was any microbiome difference between genders, but rather focused on the overall fungal microbiota composition across all patients. In the end, only 14 patients remained in the study. Additionally, the study focused exclusively on fungal microbiota without incorporating bacterial interactions; this might overlook crucial microbiome dynamics that could influence psoriasis outcomes. The cross-sectional design, analyzing data at specific time points, restricts insights into long-term microbial changes and their effects on disease progression or treatment response to brodalumab. Potential biases in sample processing, such as variability in DNA extraction efficiency or bead-beating impact, could also affect data reliability, especially because the focused ITS variation is not as pronounced in fungal species, making identification difficult. Lastly, the study’s emphasis on taxonomic classification without exploring functional roles of the fungi limits the understanding of their contributions to disease mechanisms. 

Future research could address these issues by including a larger and more diverse cohort, analyzing possible gender differences in fungal composition, integrating both fungal and bacterial microbiota, employing a longitudinal approach to track changes over time, and thus incorporating functional analyses. Additionally, future research will leverage nanopore long-read sequencing technology to produce longer DNA sequences that cover the ITS region and Large subunit ribosomal (LSU) rRNA gene within the fungal rRNA operon [28]. By focusing on the fungal rRNA operon, we aim to overcome the limitations of this study by minimizing the risk of contaminating plant-derived sequencing reads. Overall, these advancements will enable a more comprehensive understanding of the microbiome’s influence on psoriasis and treatment efficacy and pave the way for more targeted and effective treatment strategies.

## 4. Materials and Methods

This study is part of a pilot project by Andersch-Björkman et al. (2023) and Vižlin et al. (2024), and it follows the same study design [17,29].

### 4.1. Clinical Outcome Sequencing and Evaluation

Eurofins Genomics (Konstanz, Germany) conducted the bioinformatics and sequencing. The 18S rRNA’s ITS2 region was the focus of the sequencing effort. DNA extraction was performed by Eurofins Genomics (Konstanz, Germany) using commercial kits with in-house optimized and validated protocols, including both chemical and mechanical lysis of cells.. A two-step PCR technique was then carried out. In the first stage, region-specific primers (forward: GCATCGATGAAGAACGCAGC, reverse: TCCTCCGCTTATTGATATGC [30]) were used to target the ITS2 region of the 18S rRNA. These primers included an Illumina TruSeq adapter, a universal adaptor sequence for indexing that would come later. After indexing, the samples were pooled to provide around 60,000 read pairs for each sample. These were then loaded into an Illumina MiSeq flow cell (Illumina, San Diego, CA, USA) and sequenced in 2 × 300 bp mode using v3 chemistry. FASTQ files containing sequences and quality ratings were provided [29]. At every sample point, the MADRS-S, DLQI, PASI, and HADS scores were evaluated to examine the clinical results [19,20,21]. 

### 4.2. Bioinformatics

The UCHIME de novo algorithm in the VSEARCH package version 2.3.0 was used to remove ambiguous reads and chimeric sequences. Minimum Entropy Decomposition was then used to organize the remaining reeds into OTUs. Any OTUs with an average abundance level of less than 0.5% across all samples were removed, and the remaining OTUs were processed for comparative statistical analysis. DC-MEGABLAST (version 2.9.0+) assigned taxonomic information to each OTU by aligning cluster-representative sequences with reference sequences (minimum of 70% sequence identity across at least 80% of the sample sequence). With QIIME (version 1.9.1), additional processing and taxonomic designations were carried out. To improve estimates, lineage-specific copy counts of the relevant marker genes were used to normalize abundances of fungal taxonomic units. The taxonomic assignment was finished at the lowest rank that was feasible [29].

### 4.3. Statistical Analysis

The study employed beta diversity analysis to investigate the variations in OTU compositions between treatment groups and the gut microbiota of individual patients. Bray–Curtis dissimilarity values were generated from the OTU compositions. To ascertain the statistical significance of differences between treatment groups and individual patients’ gut microbiota, PERMANOVA (a multivariate extension of ANOVA) was performed on the Bray–Curtis dissimilarity values. Pairwise PERMANOVA was employed to evaluate the significance of gut microbiota differences before and after the switch to brodalumab, considering interpatient variations. Alpha diversity analysis (Shannon–Wiener diversity index) was performed using OTU counts as input data incorporating a number of distinct OTUs and relative abundance of each OTU. Considering interpatient variances, the significance of differences between treatment groups was assessed by pairwise comparisons using the Wilcoxon signed-rank test on the alpha diversity values. All statistical analyses were carried out using the pairwise Adonis package in R version 0.4.1 and the vegan version 2.6.4, which included alpha and beta diversity calculations. PCoA involved converting OTU counts to log-ratios and then Euclidean distance metric was used to calculate sample distances. To represent the samples in a reduced-dimensional space while preserving the dissimilarity relationship between them, an ape package in R version 5.7.1 was utilized. Following the switch to brodalumab, the patterns of similarity and dissimilarity between OTUs in the gut microbiota were displayed by the visualization of the resulting coordinates in a two-dimensional PCoA plot (Figure 3a,b). To conduct Differential Abundance Analysis (DAA) and find noteworthy changes in particular taxa at the phylum, genus, and species levels, the R/Bioconductor program DESeq2 version 1.38.3 was used. Using the Benjamini–Hochberg technique, all *p*-values were adjusted for false discovery rates (FDRs), with a significance threshold of FDR < 0.1. Using the Benjamini–Yekutieli method to account for multiple testing, Spearman correlation was used to correlate the relative abundance of taxa with clinical variables (PASI, DLQI, HADS, and MADRS-S). For the correlation analysis, R version 3.5.3 (The R Foundation for Statistical Computing, Vienna, Austria) was utilized, where adjusted *p* < 0.05 is considered significant [29].

## 5. Conclusions

In conclusion, this study revealed that the fungal gut microbiota in psoriasis patients, predominantly composed of Ascomycota phylum genera such as *Saccharomyces*, *Penicillium*, *Candida*, and *Debaryomyces*, remained largely unchanged following a 12-week treatment switch to brodalumab. Despite minor changes at the genus level, the overall fungal composition remained stable, aligning with patterns seen in both psoriasis patients and healthy individuals. This stability suggests that brodalumab treatment does not disrupt the gut fungal microbiota, which is consistent with the previous study on gut bacterial microbiota [28]. While the study had some limitations, including a small sample size and a focus on fungal rather than bacterial microbiota, these findings pave the way for future research. Consideration of fungal influence is crucial in understanding the gut microbiome’s full impact by exploring both fungal and bacterial interactions. This research represents a hopeful step toward more effective, individualized care for psoriasis patients.

## Figures and Tables

**Table 1:** Patient demographics.

Variable	Sub Variable	Value
Age (years)	Mean (SD ^1^)	46.6 (11.3)
	Range (median)	30–70 (50.5)
Sex n ^2^ (%)	Female	6 (30)
	Male	14 (70)
Ethnicity n ^2^ (%)	Hispanic or Latino	1 (5.0)
	Not Hispanic or Latino	19 (95)
Mean BMI (SD ^1^)		30 (4.3)
Smokers (n ^2^)		3
First psoriasis diagnosis (age)	Mean (SD ^1^)	21.8 (10.1)
	Range (median)	11–44 (17.5)
Scalp involvement [n ^2^ (%)]	Yes	13 (65)
	No	7 (35)
Nail involvement [n ^2^ (%)]	Yes	8 (40)
	No	12 (60)
Psoriatic arthritis diagnosis n ^2^ (%)	Yes	3 (15)
	No	17 (85)
Depression diagnosis n ^2^ (%)		2 (10)
PASI	Mean (SD ^1^)	9.3 (3.5)
DLQI	Mean (SD ^1^)	10.3 (7.2)
HADS	Mean (SD ^1^)	4.3 (2.9)
MADRS-S	Mean (SD ^1^)	(6.0)

^1^ Standard deviation. ^2^ Number.

**Table 2:** Spearman correlation of phyla/genera and clinical variables.

Taxa	PASI *p*-Value (Adjusted)	DLQI *p*-Value (Adjusted)	MADRS-S *p*-Value (Adjusted)	HADS *p*-Value (Adjusted)
Ascomycota (p)	0.25 (1.00)	0.89 (1.00)	0.43 (1.00)	0.83 (1.00)
*Pencillium* (g)	0.19 (1.00)	0.18 (1.00)	0.64 (1.00)	0.41 (1.00)
*Candida* (g)	0.93 (1.00)	0.49 (1.00)	0.57 (1.00)	0.37 (1.00)
*Debaryomyces* (g)	0.38 (1.00)	0.22 (1.00)	0.98 (1.00)	0.42 (1.00)
*Saccharomyces* (g)	0.18 (1.00)	0.87 (1.00)	0.80 (1.00)	0.90 (1.00)

(p)—phylum; (g)—genus; Table showing Spearman correlation, including unadjusted and adjusted *p* values for each taxon, measured at baseline values.

## Data Availability

The original contributions presented in the study are included in the article, further inquiries can be directed to the corresponding author/s.
